# GENTRIFICATION, AGING, AND HEALTH: A NARRATIVE REVIEW

**DOI:** 10.1093/geroni/igad104.0068

**Published:** 2023-12-21

**Authors:** Dionne Bailey

**Affiliations:** University of Minnesota, Minneapolis, Minnesota, United States

## Abstract

Multiple definitions of gentrification exist, and the lack of consensus about what constitutes gentrification has limited the efforts of this concept’s measurement/operationalization as well as an understanding of how gentrification influences outcomes, particularly for older adults. The objective of this literature review is to synthesize existing definitions and measurement approaches to gentrification in studies of older adults and their communities. Another objective is to summarize the health implications of gentrification for older adults. OVID Medline, CINAHL, Academic Search Premier, PsycINFO, and Scopus were searched in order to identify peer-reviewed articles that were published up to August 2022. ‘Gentrification’ and ‘Aging’ were the keywords utilized to search for peer-reviewed articles. Post abstract screening, each article was annotated to identify key study characteristics and tabulated to identify patterns and themes. Forty-nine articles were identified and extrapolated. The analysis revealed inconsistencies in the definition of gentrification, which lead to mixed findings related to the health effects of gentrification for older people. Specifically, it was found that there are both positive and negative effects of gentrification. The most consistent findings evident were that minorities were most at-risk during periods of gentrification, particularly Black older adults. It is imperative that a scientific consensus is reached when defining and subsequently operationalizing gentrification. Such refinements will result in more robust and rigorous quantitative and qualitative research that describes the health implications and/or lived experiences of gentrification for older adults.

